# Heart rate dynamics in polyendocrine metabolic ovarian syndrome: from heterogeneity to wearable digital phenotyping

**DOI:** 10.3389/fendo.2026.1951746

**Published:** 2026-09-22

**Authors:** Tingting Ye, Junran Wang, Xiaoai Guo, Xinyue Liu, Yankui Li, Qiaofeng Wu

**Affiliations:** Acupuncture and Massage Academy, Chengdu University of Traditional Chinese Medicine, Chengdu, China

**Keywords:** autonomic dysfunction, digital phenotyping, heart rate dynamics, heart rate variability (HRV), polycystic ovary syndrome (PCOS), polyendocrine metabolic ovarian syndrome (PMOS)

## Abstract

Polyendocrine metabolic ovarian syndrome (PMOS) is the new consensus name for polycystic ovary syndrome (PCOS), emphasizing the broader multisystem nature of the condition. Altered autonomic nervous system (ANS) regulation may provide an integrative physiological perspective on the reproductive, metabolic, and cardiovascular alterations associated with PMOS/PCOS. Heart rate variability (HRV) provides a non-invasive approach for assessing autonomic regulation; however, conventional HRV indices primarily characterize the magnitude and spectral distribution of variability. We therefore propose a multidimensional framework for investigating autonomic alterations in PMOS/PCOS. It integrates conventional HRV with heart rate fragmentation, nonlinear complexity, and deceleration and acceleration capacities derived from phase-rectified signal averaging. This Review evaluates the evidence supporting these dimensions and discusses how validated wearable monitoring, standardized preprocessing, and digital phenotyping may support future context-aware autonomic research. Prospective PMOS/PCOS-specific validation is required before these approaches can inform metabolic risk stratification, treatment monitoring, or clinical decision-making.

## 1 Introduction

Polyendocrine metabolic ovarian syndrome (PMOS) was recently introduced through a multistep global consensus process as the new name for polycystic ovary syndrome (PCOS), reflecting a shift toward recognizing the condition as a broader multisystem disorder rather than solely a reproductive or gynecological syndrome1. Affecting 6%–20% of women of reproductive age worldwide (1–3), PMOS involves not only reproductive dysfunction but also profound metabolic disorders, chronic low-grade inflammation, neuropsychiatric comorbidities, and increased cardiovascular risk (2, 4–6). Although hyperandrogenism and insulin resistance are central to its pathology, they cannot fully account for the remarkable phenotypic heterogeneity or the variable cardiometabolic prognosis observed in patients (7–9). Because this terminology change is recent, the existing evidence base has largely been generated and reported under the term PCOS, and the literature therefore remains predominantly PCOS-based during this terminology transition. Accordingly, in this Review, PCOS is retained when referring to previously published evidence, whereas PMOS/PCOS is used when discussing the broader disease construct and future research framework. This combined terminology is intended to preserve continuity with the established literature while acknowledging the ongoing transition toward the new nomenclature.

Autonomic nervous system (ANS) dysregulation may represent an integrative physiological link across the reproductive, metabolic, and cardiovascular features of PMOS/PCOS. Autonomic regulation reflects the complex interactions between central neural circuits, endocrine systems, and peripheral metabolism (10–13). In PMOS/PCOS, accumulating evidence suggests that altered autonomic regulation may contribute to the links among reproductive, metabolic, and cardiovascular dysfunction (14–17). Broader mechanistic literature also supports links between autonomic signaling, insulin sensitivity, and inflammatory modulation (18, 19). These observations provide the rationale for assessing autonomic regulation in PMOS/PCOS.

HRV is a well-established, non-invasive neurophysiological marker widely utilized for the assessment of autonomic regulation. Its principal foundation rests on linear system theory, employing time-domain indices (i.e., statistical measures of interbeat interval variability such as SDNN and RMSSD) and frequency-domain indices (i.e., power spectral density components including VLF, LF, and HF bands derived from Fourier or autoregressive modeling) to quantify the statistical properties of cardiac interval series. Owing to its non-invasive nature and ease of implementation, this linear HRV framework has become a first-line tool in both clinical and research settings. Previous PCOS-focused reviews and meta-analyses have primarily characterized cardiovascular autonomic dysfunction using conventional heart rate variability (HRV), particularly time- and frequency-domain indices (14, 16). These studies generally support altered cardiovascular autonomic modulation in PCOS, although findings vary across individual indices, phenotypes, recording conditions, and analytical workflows. Conventional HRV remains valuable for characterizing the magnitude and spectral distribution of beat-to-beat variability, but it provides only a partial description of cardiac rhythm dynamics. The available PCOS literature has given comparatively less attention to complementary properties such as rhythm microstructure, broader nonlinear complexity, and direction-specific regulatory dynamics, particularly in combination with longitudinal wearable monitoring (12, 20). This leaves a conceptual and methodological gap between conventional HRV-based assessment and context-aware physiological phenotyping in PMOS/PCOS.

To bridge this gap, we propose a multidimensional heart rate dynamics framework for PMOS/PCOS that integrates conventional HRV with heart rate fragmentation (HRF), nonlinear complexity measures, and phase-rectified signal averaging (PRSA)-derived deceleration and acceleration capacities (DC/AC). These dimensions provide complementary rather than interchangeable information: conventional HRV characterizes variability magnitude and spectral distribution, HRF describes rhythm microstructure, nonlinear measures capture dynamical complexity, and PRSA-derived DC/AC characterize direction-specific deceleration and acceleration dynamics. Combined with wearable or ambulatory sensing, these complementary dimensions may extend autonomic assessment from isolated HRV measurements toward repeated, longitudinal characterization of digitally captured physiological dynamics. Such an approach may support dynamic autonomic phenotyping, in which within-person changes and context-dependent physiological patterns are evaluated alongside conventional group-level differences (20–23).

Accordingly, this Review synthesizes the available evidence relevant to these complementary dimensions and examines their differing levels of evidence maturity. It further places these dimensions within a longitudinal wearable-monitoring framework, as summarized in **Figure 1**. Given the limited direct PMOS/PCOS evidence for several of these measures, the proposed framework is intended to organize current evidence, generate testable hypotheses, and identify priorities for prospective validation rather than to imply established biomarker or clinical readiness.

**Figure 1:**
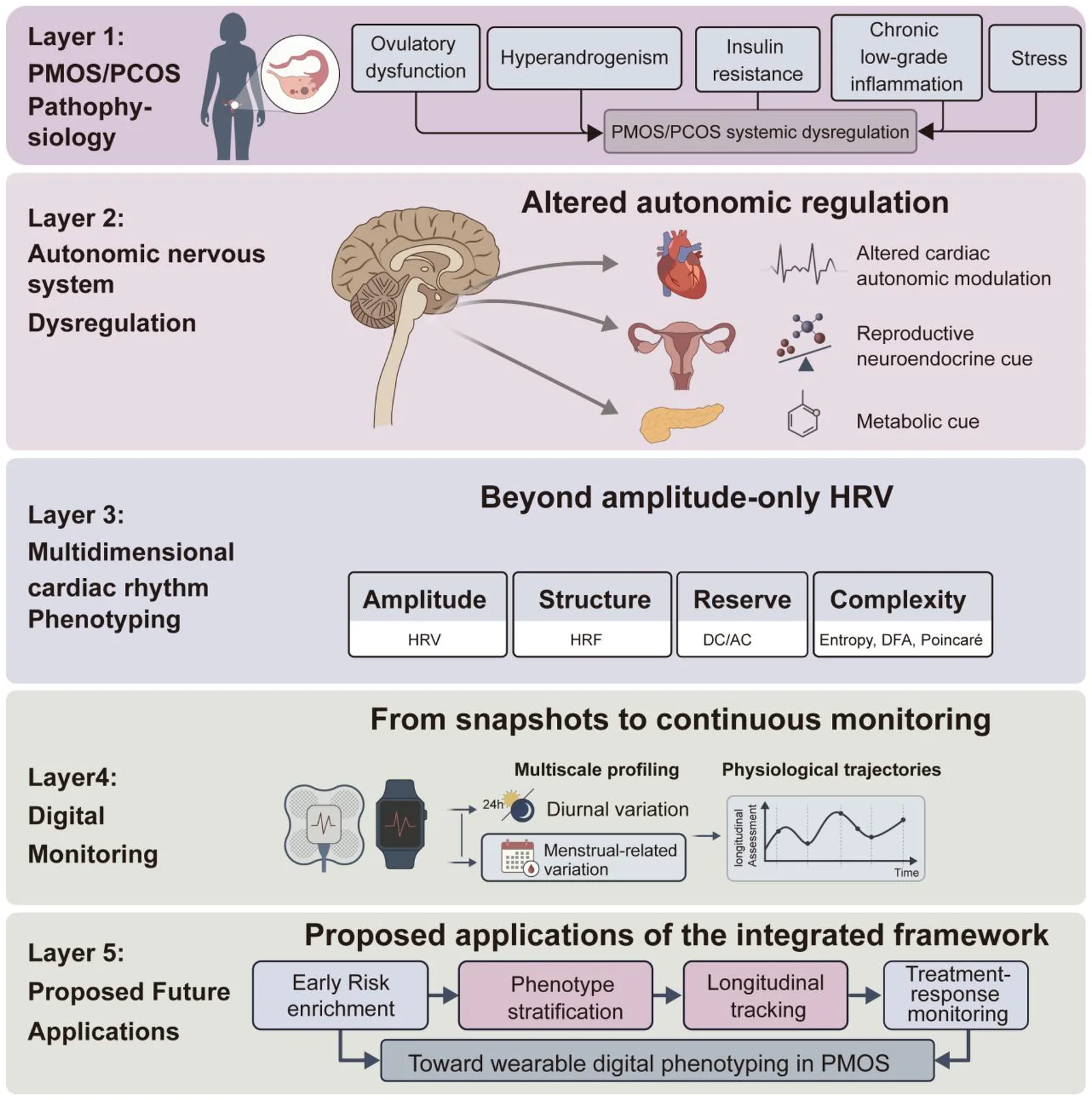
Integrated framework for multidimensional heart rate dynamics and autonomic assessment in PMOS/PCOS. Layer 1 (Pathophysiology): Key drivers including hyperandrogenism, insulin resistance, and chronic low-grade inflammation. Layer 2 (Mechanism): Illustrates altered autonomic regulation as a potential integrative link between systemic dysregulation and cardiovascular, reproductive neuroendocrine, and metabolic functional cues. Layer 3 (Physiological features): Beyond conventional HRV amplitude (SDNN), the framework incorporates rhythm microstructure (fragmentation), nonlinear complexity (entropy), and regulatory reserve (deceleration capacity). Layer 4 (Monitoring): Integration with wearable biosensors enables repeated and longitudinal assessment of digitally captured physiological dynamics across real-world contexts and physiological trajectories. Layer 5 (Proposed future applications): These multidimensional features may support future research on risk enrichment, phenotype stratification, longitudinal tracking, and treatment-response monitoring, subject to prospective validation in PMOS/PCOS.

## 2 Review approach

The primary literature search covered PubMed, Web of Science, and Embase from database inception through May 20, 2026, and was supplemented by targeted searches in Google Scholar and IEEE Xplore. Search terms covered polycystic ovary syndrome, autonomic dysfunction, heart rate variability, heart rate dynamics, heart rate fragmentation, nonlinear heart rate variability, deceleration capacity, acceleration capacity, wearable devices, digital phenotyping, and continuous monitoring. To complement the PCOS-specific literature, relevant reviews, meta-analyses, consensus statements, methodological guidelines, and selected mechanistically related studies from other populations were also considered to provide broader physiological and methodological context. Evidence was synthesized narratively without quantitative meta-analysis or formal evidence grading.

Frequently used methodological abbreviations are summarized in **Table 1**, while a more comprehensive glossary of terms and abbreviations is provided in **Supplementary Table S1**.

**Table 1:** Key methodological abbreviations used in this review.

Abbreviation	Definition
AC	Acceleration capacity
DC	Deceleration capacity
ECG	Electrocardiography
HRF	Heart rate fragmentation
HRV	Heart rate variability
IALS	Inverse average length of segments
PIP	Percentage of inflection points
PPG	Photoplethysmography
PRSA	Phase-rectified signal averaging
PSS	Percentage of short segments

## 3 Evidence and limitations of conventional HRV in PMOS/PCOS

### 3.1 Evidence linking HRV alterations to PMOS/PCOS

Preclinical and direct neurophysiological studies support sympathetic involvement in PCOS-related ovarian and metabolic dysregulation (24–28). Animal studies suggest that modulation of sympathetic pathways can affect ovarian cystic remodeling, ovulatory function, and metabolic abnormalities (24–26), while human microneurography studies have reported increased muscle sympathetic nerve activity in women with PCOS (27). However, these approaches are invasive, operator-dependent, or unsuitable for repeated monitoring, highlighting the need for scalable, noninvasive autonomic markers.

HRV therefore provides a practical bridge between autonomic physiology and digital monitoring because it is noninvasive, relatively standardized, and increasingly compatible with wearable electrocardiography (ECG) and photoplethysmography (PPG) platforms. Rather than measuring sympathetic or parasympathetic activity directly, HRV reflects integrated cardiovascular regulation shaped by autonomic, baroreflex, respiratory, and metabolic influences (29–31).

Systematic reviews and meta-analyses generally support altered cardiovascular autonomic regulation in PCOS, with relatively consistent reductions in global or vagal-related HRV indices, including SDNN, pNN50/NN50, and HF-related measures. However, findings for RMSSD, LF, LFnu, and LF/HF ratio are more variable across cohorts and protocols (14, 16), indicating that conventional HRV signals are sensitive to phenotype composition and metabolic burden (15, 32), as well as recording context and analytical workflow (31, 33–35).

This distinction is important for PMOS/PCOS because HRV-derived indices, especially LFnu and LF/HF ratio, should not be interpreted as direct measures of sympathetic tone (30, 36). Their physiological nonspecificity may partly explain inconsistent findings and limits their interpretation as stand-alone disease-specific or digital biomarkers (14, 16). **Table 2** summarizes the physiological anchors, assumptions, confounders, and interpretative boundaries of conventional HRV indices, setting the rationale for complementary analyses of rhythm organization, nonlinear complexity, and regulatory reserve.

**Table 2:** Conventional HRV indices in PCOS: physiological anchors, common findings in PCOS, key assumptions, major confounders, and interpretative boundaries.

Metric	Physiological anchor	Common findings in PCOS	Key assumptions	Major confounders	Interpretative constraints
SDNN	Global NN variability amplitude; duration-dependent	Generally reduced in PCOS; though some studies show no difference (14, 37)	Report duration; low artifacts; quasi-stationary window	BMI; IR; duration; segmentation; activity; sleep	Limited specificity for rhythm organization or regulatory capacity; recording-length dependent comparability
RMSSD	Resting, predominantly vagal short-term variability	Generally lower in PCOS; however, some trials find no difference (14, 38)	Standardized rest/posture; artifact control; stable/recorded respiration	BMI; IR; respiration; stress; sleep; blood pressure	Limited sensitivity to rhythm microstructure or regulatory reserve beyond resting vagal modulation
LF/LFnu	Composite low-frequency oscillations	Early case–control studies reported elevated LF and LFnu; later meta-analyses found inconsistent changes (14, 39)	Specify spectral method/window; nonstationarity handling	Posture; respiration; analysis settings	Limited incremental information beyond RMSSD; estimate stability reduced with noise/artifacts
HF/HFnu	Respiration-coupled vagal modulation	Often decreased in PCOS; though some studies report no difference (38, 40)	Control/record respiration; stable, high-quality segments	Respiration; sleep; motion; SQI	Not a pure vagal marker; strongly respiration- and protocol-dependent
LF/HF ratio	Ratio of two composite bands	Elevated in some early case–control reports; inconsistent in meta-analyses and MSNA recordings (37, 39)	Robust LF and HF estimation; protocol consistency	Respiration; posture; windowing	Context-dependent due to strong respiratory influence; cross-study comparability requires respiratory information

BMI, body-mass index; HF, high frequency; HFnu, normalised high-frequency power; IR, insulin resistance; LF, low frequency; LFnu, normalised low-frequency power; MSNA, muscle sympathetic nerve activity; NN, normal-to-normal; SQI, signal quality index.

Common findings are based on representative PCOS-specific studies and available systematic reviews and meta-analyses; major confounders refer to methodological and physiological factors known to influence HRV estimates and cross-study comparability.

### 3.2 Sources of heterogeneity and confounding factors in PCOS

Although conventional HRV studies generally support altered cardiovascular autonomic modulation in PCOS, findings remain heterogeneous across indices and study designs. This heterogeneity should be interpreted not simply as conflicting evidence, but as the combined effect of biological phenotype, physiological state, and analytical workflow.

First, PCOS is biologically heterogeneous. Ovulatory status, hyperandrogenism, obesity, insulin resistance, inflammation, and psychological stress may each modify autonomic regulation and alter the magnitude or direction of HRV abnormalities (7, 9). Cohorts that combine obese, insulin-resistant, anovulatory, and lower-risk phenotypes may therefore dilute or obscure phenotype-specific autonomic patterns (32). Second, HRV is highly context-dependent. Recording posture, respiration, activity, medication use, sleep-wake state, psychological stress, and menstrual-cycle phase can all influence HRV estimates (34, 35, 41). In PCOS, where sleep disturbance, menstrual irregularity, metabolic dysregulation, and psychological symptoms frequently coexist (2, 11, 42), sparse or context-insensitive recordings may miss biologically meaningful autonomic variation (41, 43, 44). This context dependence is particularly relevant to repeated and wearable-based monitoring, in which physiological state and recording conditions may vary substantially across time. Third, analytical workflow further amplifies heterogeneity. Studies differ in recording duration, device type, sampling quality, posture control, respiratory documentation, artifact correction, ectopic beat handling, interpolation strategy, segmentation length, and spectral estimation method (45, 46). Therefore, comparisons based on short-term HRV may partly reflect differences in recording context or preprocessing rather than disease-related autonomic physiology alone.

Accordingly, HRV abnormalities in PCOS should be interpreted as context-dependent signals of integrated cardiovascular autonomic modulation rather than fixed disease-specific biomarkers. These issues do not diminish the value of conventional HRV; rather, they define the conditions under which HRV should be acquired, processed, and interpreted.

### 3.3 Conceptual boundaries and interpretative constraints of conventional HRV in PCOS

Beyond between-study heterogeneity, conventional HRV metrics have intrinsic interpretative boundaries. HRV is an indirect marker of integrated cardiovascular autonomic modulation rather than a direct measurement of isolated sympathetic or parasympathetic neural activity (31). Accordingly, LF, LFnu, and LF/HF ratio should not be interpreted as one-to-one indicators of sympathetic tone or sympathovagal balance (36, 47). This distinction is particularly relevant in PMOS/PCOS, where endocrine, metabolic, psychological, sleep-related, and reproductive contexts may jointly shape heart rate dynamics.

Conventional time- and frequency-domain HRV indices quantify the amount and spectral distribution of NN-interval variability, but provide limited information about how this variability is dynamically organized (48). Similar SDNN, RMSSD, or HF values may conceal different rhythm patterns, and reduced HRV may arise from different physiological contexts with different clinical implications (49). Conventional HRV therefore remains useful for standardized assessment of integrated autonomic modulation, but it is insufficient on its own to characterize rhythm organization, nonlinear complexity, and dynamic regulatory reserve. These limitations support extending HRV assessment toward complementary multidimensional features that may be captured repeatedly across time. **Table 3** summarizes these conceptual limitations and the complementary analytic dimensions developed in the next section.

**Table 3:** Conceptual limitations of conventional HRV in PCOS and corresponding complementary analytic dimensions.

Limitation of conventional HRV	Potential interpretative bias in PCOS studies	Complementary analytic dimension
Quantifies variability magnitude, not rhythm organization	Apparent normality: preserved amplitude despite altered rhythm organization	Rhythm microstructure assessment
Relies on linearity and quasi-stationarity assumptions	Hormonal fluctuation, metabolic load, and sleep disruption increase nonstationarity; short-term HRV may underestimate dysregulation	Nonlinear complexity assessment
Limited mechanistic specificity	Reduced HRV may reflect transient stress vs chronic autonomic impairment, with divergent clinical meaning	Integrated structure–complexity assessment
Lacks assessment of regulatory reserve	Resting HRV may miss impaired reactivity/recovery and early autonomic vulnerability	Regulatory reserve assessment
Constrained by short, static windows	PCOS may show diurnal and menstrual-cycle–related rhythm modulation; single snapshots miss trajectories	Longitudinal dynamics assessment

## 4 Beyond amplitude: rhythm microstructure, nonlinear complexity, and dynamic regulatory reserve

The following sections develop three complementary dimensions of heart rate dynamics beyond conventional amplitude- and spectral-power metrics. Heart rate fragmentation (HRF) characterizes rhythm microstructure and beat-to-beat discontinuity; nonlinear HRV indices assess complexity, irregularity, and scale-dependent organization; and phase-rectified signal averaging (PRSA)-derived deceleration capacity (DC) and acceleration capacity (AC) quantify direction-specific regulatory reserve.

Importantly, the evidence supporting these dimensions differs in maturity. Conventional HRV has been directly investigated in PCOS across multiple clinical studies and systematic reviews, whereas direct PMOS/PCOS-specific evidence for HRF, many nonlinear HRV measures, and PRSA-derived DC/AC remains limited. The PCOS-specific conventional HRV literature is itself constrained by generally modest sample sizes, predominantly observational study designs, and methodological heterogeneity in phenotype definition, recording conditions, preprocessing, and metric selection. Accordingly, evidence from cardiovascular, metabolic, neurological, aging, and stress-related populations is used here as a physiological and methodological basis for future investigation rather than as proof of established effects in PMOS/PCOS. The relative evidence maturity, key limitations, and current interpretation of these approaches are summarized in **Table 4**.

**Table 4:** Evidence maturity of heart rate dynamics and digital phenotyping in PMOS/PCOS.

Dimension	Direct PMOS/PCOS evidence	Supporting evidence from other populations or experimental models	Key limitations/confounders	Current interpretation in PMOS/PCOS
Conventional HRV	Relatively established	Extensive	Phenotype; BMI/IR; respiration; recording context	Well-established research measure, but context-dependent
HRF	Limited	Cardiovascular disease, aging, T2D, CAC, cerebral small vessel disease	Signal quality; artifacts; recording duration	Candidate/hypothesis-generating measure
Nonlinear HRV	Limited	Diabetic autonomic neuropathy, experimental metabolic syndrome, psychological stress	Data length; parameter choice; nonstationarity	Candidate/hypothesis-generating measure
PRSA-derived DC/AC	Limited	Post-MI, cerebrovascular disease, cardiomyopathy, AF, anesthesia and general populations	Signal quality; parameter settings; algorithm dependence	Candidate/hypothesis-generating measure
Wearable ECG/PPG	Limited PCOS-specific validation	Extensive wearable and digital health literature	Motion; signal quality; device variability	Research platform for repeated monitoring
Longitudinal digital phenotyping	Very limited	Emerging wearable and multimodal literature	Context; missing data; device heterogeneity	Conceptual framework requiring prospective validation

AF, atrial fibrillation; BMI, body mass index; CAC, coronary artery calcification; ECG, electrocardiography; IR, insulin resistance; MI, myocardial infarction; PPG, photoplethysmography; T2D, type 2 diabetes.

Against this background, HRF, nonlinear HRV measures, and PRSA-derived DC/AC should be regarded as complementary research measures rather than established PMOS/PCOS biomarkers. Their potential value lies in capturing aspects of cardiac rhythm dynamics that are not fully represented by conventional HRV. Because these measures can be derived from digitally acquired cardiac interval series, they may also be suitable for repeated and longitudinal assessment, provided that adequate signal quality and analytical standardization are ensured. Detailed definitions, formulas, and computational procedures for these metrics are summarized in **Supplementary Table S2**.

### 4.1 Heart rate fragmentation

HRF addresses a question that conventional time- and frequency-domain HRV indices do not fully capture: whether beat-to-beat rhythm remains temporally organized. Whereas conventional HRV mainly quantifies the amount or spectral distribution of NN-interval variability, HRF characterizes microstructural discontinuity in the NN-interval series, including frequent directional changes and shortened acceleration or deceleration runs (49). In this sense, HRF is better viewed as a descriptor of altered rhythm organization than as a direct surrogate of sympathetic or parasympathetic tone (**Figure 2**).

**Figure 2:**
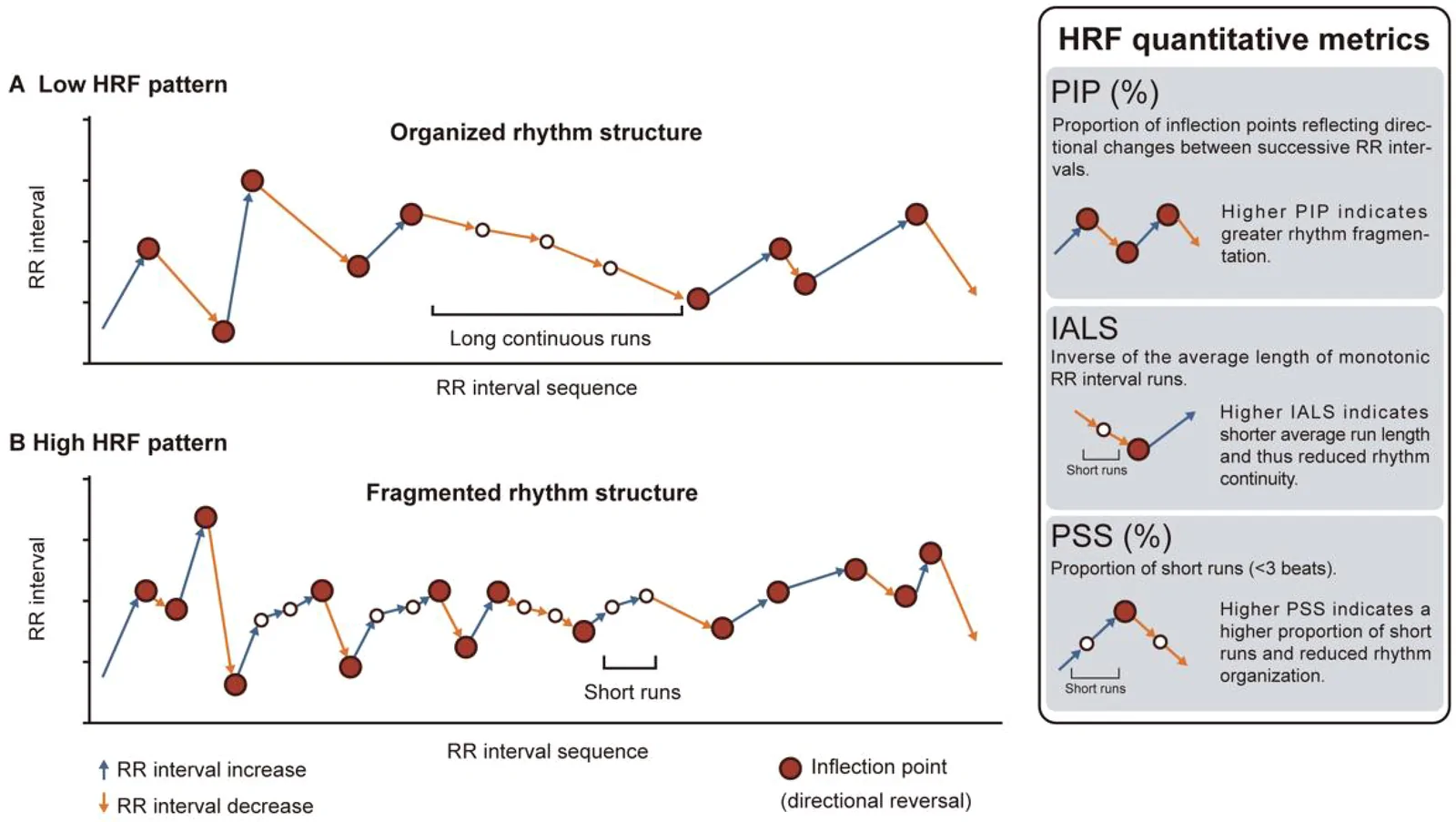
Schematic illustration of HRF based on NN-interval directional changes. HRF quantifies the degree to which NN-interval dynamics are disrupted by frequent directional reversals in the NN-interval time series. **(A)** Low HRF is characterized by a more organized rhythm structure and longer continuous runs. **(B)** High HRF is characterized by more frequent directional reversals and shorter runs. PIP, IALS, and PSS summarize complementary aspects of rhythm fragmentation, including directional reversals, inverse average segment length, and the proportion of short runs. Its applicability in PMOS/PCOS requires further validation.

Evidence from cardiovascular, aging, and metabolic disease research suggests that elevated HRF may provide information partly independent of conventional HRV metrics (50–52). In type 2 diabetes mellitus, HRF analysis has shown increased fragmentation patterns compared with healthy controls, suggesting that HRF may capture heart rate dynamic abnormalities in a metabolic disease context (52). Higher HRF has also been associated with subclinical target-organ damage, including coronary artery calcification and cerebral small vessel disease, even when amplitude-based HRV indices show limited or no differences (53, 54). These findings suggest that rhythm fragmentation may reveal altered temporal organization of cardiac control that is not captured by variability magnitude alone.

However, direct evidence evaluating HRF in PMOS/PCOS remains limited, and its incremental value beyond conventional HRV has not yet been established in this population. Extending this structural perspective to PCOS is physiologically plausible. PCOS is commonly accompanied by chronic low-grade inflammation, insulin resistance, and hyperandrogenism, factors that may alter neuroendocrine and autonomic regulatory coupling (12, 16). These observations therefore raise the testable hypothesis that HRF may provide complementary information to conventional HRV when overall variability appears relatively preserved but beat-to-beat rhythm organization is disrupted. Future studies in PMOS/PCOS should test whether HRF identifies such rhythm-organization phenotypes after accounting for phenotype, metabolic status, sleep, menstrual-cycle context, and preprocessing workflow.

### 4.2 Nonlinear complexity indices

Nonlinear HRV approaches address a second question that conventional amplitude- and spectral-power–based indices do not fully answer: whether heart-rate dynamics exhibit altered irregularity, correlation structure, and scale-dependent organization over time. These methods characterize properties such as irregularity, local correlation structure, and scale-dependent organization, and may be informative when conventional HRV magnitude is similar but the temporal structure of beat-to-beat dynamics differs (55, 56). Common approaches include Poincaré plot–derived geometry, entropy-based measures, and fractal or scale-correlation analyses such as detrended fluctuation analysis. Poincaré-derived indices summarize short- and long-term beat-to-beat dispersion; entropy measures quantify irregularity and multiscale complexity; and fractal scaling approaches assess whether fluctuations remain correlated and organized across temporal scales.

Evidence from metabolic and stress-related populations suggests that nonlinear HRV indices can capture information not conveyed by variability amplitude alone. In diabetic autonomic neuropathy, altered entropy and Poincaré-derived measures have shown potential value beyond traditional HRV metrics, including for asymptomatic autonomic involvement (57). Studies of experimental metabolic syndrome and psychological stress have similarly reported alterations in entropy, geometric parameters, and fractal scaling properties (58, 59).

Direct PMOS/PCOS-specific evidence for many of these nonlinear measures remains limited, and their added value beyond conventional HRV has not been established in this population. Nevertheless, because PCOS involves interacting metabolic, endocrine, and autonomic disturbances, extending this complexity-based perspective to PMOS/PCOS is physiologically plausible, but should currently remain hypothesis-generating. Future studies should test whether nonlinear HRV measures identify alterations in irregularity, correlation structure, or scale-dependent organization that are not fully captured by conventional HRV. Their estimates depend on recording length, data quality, preprocessing strategy, parameter selection, and the handling of nonstationarity; therefore, standardized acquisition and analytic protocols remain essential for reliable cross-study comparison (60–63).

### 4.3 Deceleration capacity and acceleration capacity

Beyond rhythm structure and complexity, PRSA-derived deceleration capacity (DC) and acceleration capacity (AC) address a third question: whether cardiac autonomic control retains direction-specific regulatory reserve. PRSA identifies acceleration- or deceleration-related anchor points within NN-interval series, aligns the surrounding segments, and averages them so that recurring regulatory patterns can be detected despite noise and nonstationarity (64, 65). This approach differs conceptually from conventional HRV in two respects: it separately characterizes deceleration- and acceleration-related dynamics and focuses on regulatory responsiveness rather than variability magnitude alone. DC is commonly interpreted as reflecting vagally mediated deceleration capacity, whereas AC quantifies acceleration-related dynamics within the same PRSA framework (64–66). Unlike heart rate turbulence, PRSA does not require ventricular premature beats as triggers, which may improve its feasibility in short or clean sinus-rhythm recordings. Their conceptual role is summarized in **Figure 3**.

**Figure 3:**
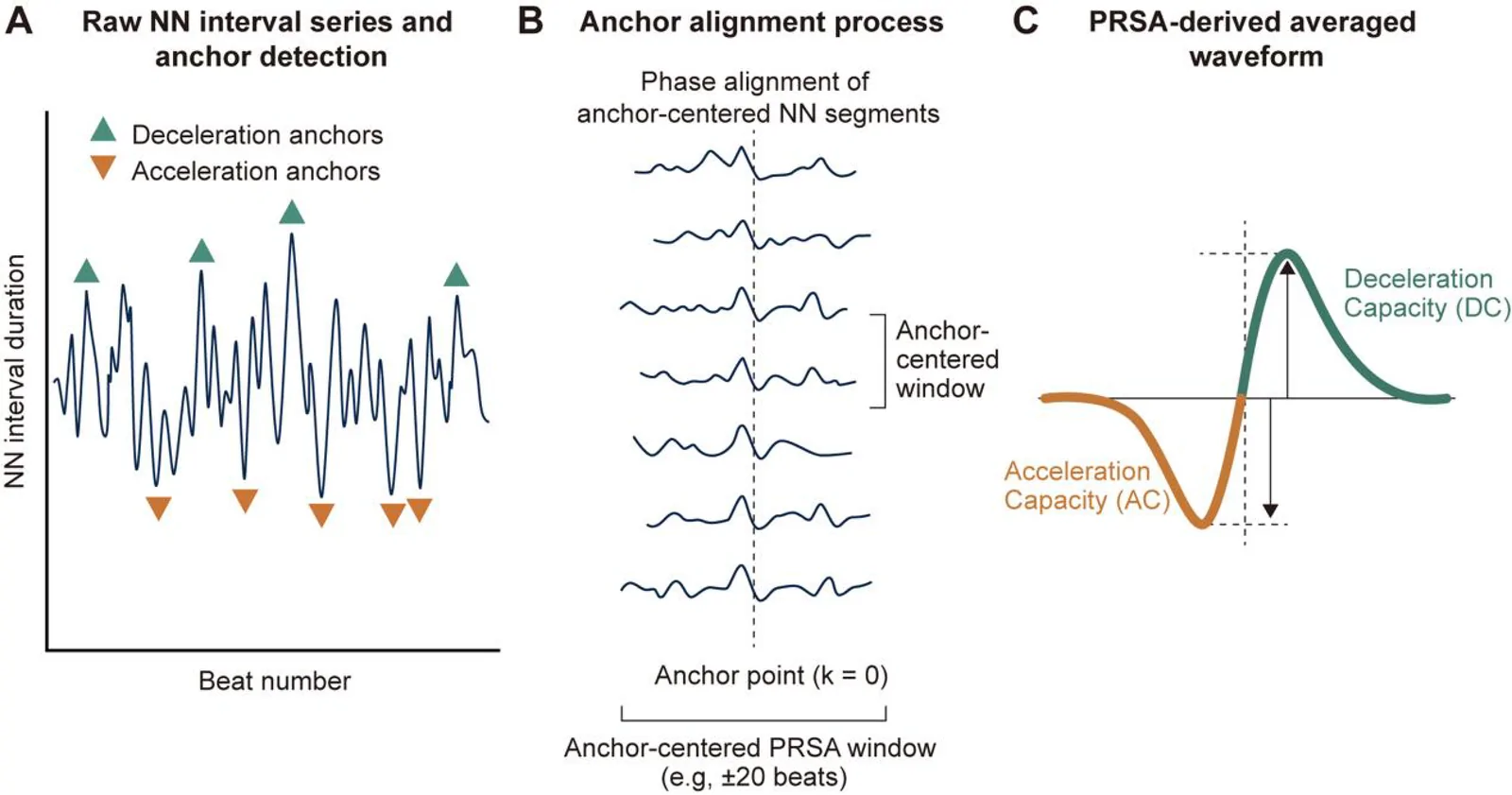
Schematic illustration of PRSA-derived DC and AC. **(A)** Raw NN interval series with identification of acceleration and deceleration anchors. **(B)** Phase alignment of anchor-centered NN segments within the PRSA window. **(C)** PRSA-derived averaged waveform used to derive deceleration and acceleration capacity. Its applicability in PMOS/PCOS requires further validation.

Evidence from non-PCOS populations supports DC and AC as markers of regulatory reserve. In post-myocardial infarction populations, DC has been shown to outperform conventional HRV indices and left ventricular ejection fraction in predicting mortality risk (65). Lower DC has also been associated with poorer functional recovery after cerebrovascular disease (67), and DC or AC has been investigated in structural heart disease, arrhythmia-related conditions, and perioperative risk assessment (66, 68–70). Methodological studies further indicate that optimized PRSA algorithms can improve the stability and discriminative performance of DC in complex clinical signals (71). DC has shown prognostic value in a general-population study, supporting its feasibility for standardized short-window assessment (72). However, direct PMOS/PCOS-specific evidence for DC and AC remains limited. Nevertheless, given the reported alterations in autonomic regulation in PCOS, extending direction-specific assessment of cardiac acceleration and deceleration dynamics to this population is physiologically plausible. DC and AC may therefore serve as candidate measures for testing whether PCOS-related autonomic dysregulation involves altered deceleration and/or acceleration dynamics that are not fully captured by conventional variability magnitude. This hypothesis requires direct validation in PMOS/PCOS.

Taken together, these three analytic domains extend autonomic assessment beyond variability amplitude by capturing rhythm structure, dynamical complexity, and regulatory reserve. Because these features are often state-dependent and temporally heterogeneous, their interpretation benefits from longer or repeated recordings rather than brief resting snapshots alone.

## 5 Translating multidimensional heart rate dynamics into clinical practice

Translating multidimensional heart rate dynamics into clinically usable tools requires more than defining analytic metrics; it also depends on whether these metrics can be acquired, processed, and interpreted reproducibly despite biological and methodological heterogeneity in PCOS. This process requires three linked components: continuous data acquisition, standardized signal-processing workflows, and clinically interpretable implementation pathways. Wearable and remote monitoring may support this transition by enabling repeated assessment of heart rate dynamics across PCOS phenotypes, metabolic states, circadian timing, behavioral contexts, and menstrual-cycle phases, thereby providing a basis for longitudinal digital physiological phenotyping.

### 5.1 Wearable acquisition: ECG and PPG as complementary data sources

Wearable monitoring extends autonomic assessment from isolated laboratory recordings to repeated or continuous physiological observation in real-world settings. This approach is particularly relevant to PMOS/PCOS because autonomic regulation may vary across sleep, activity, circadian timing, and reproductive hormone-related contexts (31, 32, 41, 73). Longitudinal profiling may help distinguish persistent autonomic patterns from transient fluctuations driven by behavior, cycle phase, or measurement timing, thereby improving the interpretation of heterogeneous HRV findings (21, 73). Current wearable approaches can be broadly grouped into patch-based or chest-worn ECG platforms and wrist-worn PPG platforms (**Figure 4**).

**Figure 4:**
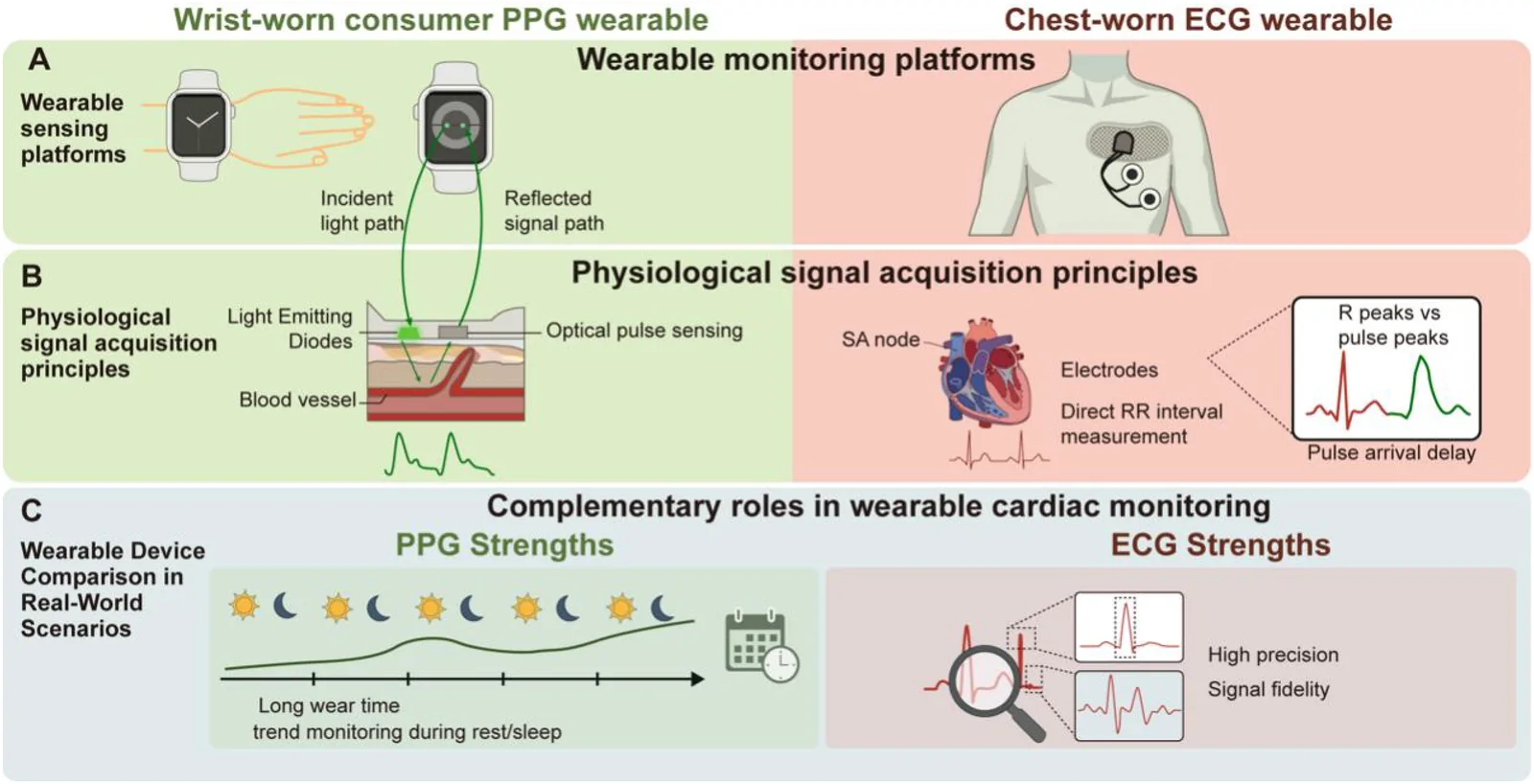
Complementary roles of wrist-worn PPG and chest-worn ECG in wearable cardiac monitoring. PPG supports long-duration, high-compliance continuous monitoring, whereas ECG enables direct detection of cardiac electrical activity and precise derivation of RR intervals, with higher temporal precision and signal fidelity. Panels **(A–C)** illustrate device platforms, sensing principles, and their complementary strengths in real-world monitoring. ECG and PPG are presented as monitoring platforms requiring PMOS/PCOS-specific validation.

Device selection should follow the measurement purpose. ECG directly records cardiac electrical activity and remains the reference standard for accurate NN-interval extraction, making patch-based or chest-worn ECG preferable when studies require precise beat-to-beat interval estimation for HRV and higher-order heart rate dynamics analyses (34, 48). Single-lead ECG patches can support continuous recordings over 24–72 h or longer and may complement conventional Holter monitoring, making them suitable for mechanistic studies, protocol-driven phenotyping, and treatment-response assessments (74, 75). However, during extended monitoring periods, such as multi-week follow-up or assessments spanning menstrual cycles, device adherence and wearing burden may limit feasibility. Accordingly, wearable ECG is particularly suited to shorter-term, high-fidelity phenotyping assessments.

Wrist-worn PPG may be useful for low-burden longitudinal monitoring during daily life and sleep, particularly for characterizing diurnal, sleep-related, or menstrual-cycle-related patterns in PMOS/PCOS. However, PPG-derived heart rate and interbeat intervals are inferred indirectly from peripheral blood-volume changes and are susceptible to motion artifacts and variations in signal quality (76). ECG should therefore remain the reference standard for validating PPG-derived mean heart-rate estimates (77). However, this validation does not extend to PPG-derived interbeat intervals, HRV, or higher-order heart-rate dynamics (78).

In PMOS/PCOS studies, a practical workflow is to use ECG for high-fidelity validation and PPG for scalable longitudinal digital phenotyping. In addition to ECG- or PPG-derived heart rate signals, contemporary wearable systems often integrate accelerometers and other sensors that provide contextual information, including respiratory rate, sleep, activity level, motion, and estimated energy expenditure. These variables can be treated as contextual covariates that help distinguish autonomic changes from behavioral or physiological confounding and improve the reproducibility of longitudinal autonomic phenotyping.

Despite these advantages, practical limitations should be considered when deploying wearable devices for longitudinal PMOS/PCOS monitoring. Motion artifacts, signal-quality variation, sensor drift, and battery limitations may affect data completeness and reliability (79). Device-specific or proprietary algorithms can further limit transparency and cross-device comparability (21, 23). In addition, continuous physiological monitoring raises considerations regarding data privacy, cybersecurity, and regulatory oversight (80). These factors should therefore be addressed when designing and validating wearable-based autonomic phenotyping studies.

### 5.2 Digital phenotyping framework and standardization

#### 5.2.1 Signal preprocessing

In long-term continuous monitoring datasets, reliable characterization of autonomic digital phenotypes depends on preprocessing pipelines that are sufficiently standardized to preserve both data quality and analytical comparability. Given the inherently nonstationary nature of heartbeat interval time series under real-world conditions, data processing should first identify appropriate recording periods and account for motion artifacts, baseline drift, and variability in signal quality (48). Signal quality indices can further help exclude unstable ECG or PPG segments before structural and complexity-based feature extraction (81). Detrending procedures are commonly recommended in heart rate analysis to minimize the effects of low-frequency drift, with frequently used approaches including smoothness priors–based detrending algorithms. Detection and correction of ectopic or abnormal beats may further improve the reliability of HRV estimation (82). Artifact-related disturbances can be addressed using outlier detection combined with cubic spline interpolation to preserve the continuity of R–R interval series (83). Together, these steps provide the technical basis for reproducible multidimensional heart rate feature extraction and physiologically interpretable digital phenotyping.

#### 5.2.2 Standardized implementation workflow in PMOS/PCOS

To improve reproducibility and cross-study comparability, relatively standardized procedures are needed for data acquisition, signal quality control, preprocessing, feature extraction, and reporting in longitudinal digital monitoring studies of PCOS. Based on current heart rate dynamics research and wearable monitoring practice, a reference workflow for implementation is summarized in **Table 5**.

**Table 5:** Methodological considerations for wearable heart rate dynamics monitoring in PMOS/PCOS.

Assessment domain	Technical recommendation	Relevance
Hardware and sampling	ECG preferred for beat-to-beat HRV and higher-order dynamics; PPG requires signal-quality assessment and ECG validation where feasible	Reliable NN-interval estimation and cross-device comparability
Monitoring duration	Match recording duration to the target metric and biological timescale; use ≥24 h when diurnal profiling is intended, and standardized rest or sleep windows for shorter protocols.	Capture diurnal variation and improve protocol comparability
Motion and activity annotation	Use accelerometer data to identify rest, sleep, low-activity, and high-motion segments	Reduce activity-related confounding and support context-specific interpretation
Respiration and sleep context	Record sleep/wake state and breathing rate or respiratory proxies where feasible	Reduce respiratory and state-dependent confounding, especially for frequency-domain HRV
Artifact handling and detrending	Apply validated artifact correction and appropriate detrending; preserve interval continuity when possible	Reduce analytical bias and improve feature stability
Reproductive context annotation	Record menstrual-cycle phase, bleeding pattern, and hormonal treatment status where available	Reduce cycle-related confounding in longitudinal interpretation
Participant characterization	Record PCOS phenotype, BMI or obesity status, insulin resistance, androgen status, ovulatory status, medication use, and relevant comorbidities	Reduce phenotype-related heterogeneity and support subgroup interpretation

### 5.3 Clinical applications and translational value

Multidimensional heart rate dynamics have the potential to contribute to PMOS/PCOS research and future clinical translation in three areas: risk enrichment, phenotype stratification, and longitudinal treatment monitoring. First, these metrics may complement conventional PCOS phenotyping based on reproductive and endocrine features. They may identify autonomic alterations that are not fully captured by traditional metabolic or reproductive markers, such as increased sympathetic nerve activity (27) or phenotype-related differences in cardiac autonomic modulation (32). Given prospective cohort data (5) and systematic evidence (6) suggesting that cardiovascular risk in women with PCOS may increase relatively early in life, these candidate autonomic patterns may eventually provide complementary information for risk characterization.

Second, standardized wearable monitoring enables HRF, nonlinear indices, and PRSA-derived measures to characterize inter-individual variation in autonomic regulation across metabolic, reproductive, sleep, and behavioral contexts. They may also serve as candidate digital physiological markers for quantifying autonomic changes and supporting future studies of treatment-related responses (21, 22, 84). Such applications would be most informative when changes in these metrics are analyzed together with PCOS phenotype, BMI or obesity status, insulin resistance, hyperandrogenism, ovulatory status, and medication exposure.

Third, longitudinal follow-up shifts autonomic assessment from isolated measurements to intra-individual dynamic trajectories. Menstrual-cycle phase, bleeding pattern, and hormonal treatment status should be recorded because HRV varies systematically across reproductive hormonal states (44, 85). Continuous wearable monitoring can contextualize autonomic trajectories within cycle-related physiological variation and reduce misclassification from isolated measurements. This longitudinal perspective supports more individualized follow-up in PCOS, although prospective validation is needed before these metrics can be used for clinical decision-making.

## 6 Future directions for clinical translation

### 6.1 Interoperability and cross-device validation

The wearable device ecosystem remains highly fragmented, with substantial differences across manufacturers in data formats, signal processing pipelines, and data access permissions, thereby limiting the integrated use of continuous physiological data (86). Differences in signal structure across monitoring settings, from hospital-based 12-lead ECG and ICU multimodal monitoring to home-based single-lead ECG or PPG, further restrict transferability, because many existing analytical approaches remain tailored to specific input formats and use contexts. Device-agnostic integration models, such as the Basel Wearable Clinic, illustrate the need for standardized upload pathways, secure data handling, and expert review of wearable ECG data (87).

For PMOS/PCOS digital phenotyping, the key technical priority is cross-device comparability. Future frameworks should define shared reporting standards for device type, sampling rate, signal quality, preprocessing, artifact correction, and feature extraction. Standardized data models based on Open mHealth and HL7 FHIR may facilitate the interoperable exchange of wearable data (88). Before clinical interpretation, wearable-derived HRV and heart-rate dynamics features should be validated against the corresponding features derived from standardized ECG recordings.

### 6.2 Longitudinal validation across biological timescales

PMOS/PCOS-related autonomic regulation should be evaluated across biologically relevant timescales rather than only through isolated resting recordings. Circadian rhythms, sleep–wake states, menstrual-cycle phases, reproductive hormone profiles, and metabolic fluctuations can all shape heart rate dynamics (32, 73, 89). This is particularly important because short-term HRV recordings can support standardized group-level comparisons, whereas individualized characterization of autonomic regulation may require broader temporal and contextual information (31, 90).

Longitudinal wearable monitoring can help characterize whether altered heart rate dynamics represent persistent patterns, transient physiological fluctuations, or treatment-related changes. Future studies should define sampling windows that align with circadian, sleep-related, reproductive, and metabolic timescales, and should report cycle phase, bleeding pattern, hormonal therapy, sleep, activity, and metabolic status when interpreting repeated HRV and heart rate dynamics measurements.

Validation should extend beyond single-centre studies to multicentre cohorts with prespecified external validation. Reproducibility should be assessed across independent datasets, devices, analytical pipelines, and populations with diverse demographic and metabolic characteristics. Longitudinal studies should additionally report follow-up duration, sampling frequency, adherence, missingness, and the handling of time-varying contextual factors. Where feasible, appropriately governed data sharing and transparent analytical workflows should be encouraged to support independent replication and cross-population validation.

### 6.3 Dynamic confounding, multimodal integration, and AI-enabled digital phenotyping

Autonomic regulation in women with PCOS is shaped by behavioral, metabolic, endocrine, and environmental factors (20). Interpreting a single heart rate dynamics metric without context can overestimate differences driven by acquisition conditions or behavior and underestimate clinically meaningful pathological variation (23). Context-aware digital phenotyping should therefore integrate heart rate dynamics with sleep architecture, physical activity, motion, respiratory information, glucose patterns, medication exposure, and cyclical endocrine information.

This multimodal approach may help distinguish disease-related autonomic patterns from context-driven physiological fluctuations. Recent feasibility data on continuous glucose monitoring in PCOS provide an entry point for linking metabolic variability with autonomic regulation (91). As wearable systems evolve toward multimodal physiological and molecular sensing, future models should prioritize cross-modal alignment, interpretable modeling, and prespecified validation (92). After longitudinal validation, these approaches could support composite digital phenotypes for context-aware risk enrichment in PMOS/PCOS.

Machine-learning approaches have already been explored in PCOS, including prediction using large electronic health record datasets (93). More broadly, within the multimodal framework outlined above, AI-based approaches may facilitate the integration of heart rate dynamics with metabolic, reproductive, behavioral, and wearable-derived contextual data (94). Such approaches could support autonomic phenotype classification and predictive modeling by identifying complex, potentially nonlinear relationships across these data streams. Interpretable multimodal models may further help clarify which physiological features contribute to individual variation (92, 94). However, AI-enabled applications remain largely exploratory in PCOS, and their incremental value over conventional analytical approaches has yet to be established. Future studies should therefore evaluate model calibration, interpretability, external validity, cross-device generalizability, and clinical utility before their use for individualized risk or treatment-response prediction can be considered.

### 6.4 Implementation and real-world considerations

Beyond technical and clinical validation, translating wearable-based autonomic phenotyping into practice will require attention to broader health-system and real-world factors. Future studies should assess cost-effectiveness and the infrastructure required for data acquisition, storage, processing, and clinical interpretation. Clinical implementation will also require appropriate clinician training and integration with electronic health record systems and existing clinical workflows. Accessibility and affordability should also be addressed to minimize the risk of widening disparities, particularly in resource-constrained settings. These implementation considerations should be evaluated alongside technical performance and clinical utility before routine clinical adoption.

## 7 Conclusion

In summary, multidimensional heart rate dynamics provide a potentially informative framework for characterizing heterogeneity in autonomic regulation in PMOS/PCOS. Wearable monitoring could extend this framework by capturing temporal variability across physiological and behavioral contexts beyond isolated measurements. Integrating high-resolution heart rate dynamics with endocrine and metabolic biomarkers may support a more continuous and physiology-informed characterization of disease trajectories and treatment-related changes. However, the clinical relevance and incremental value of these approaches remain to be established. Future prospective and longitudinal studies should determine whether context-aware autonomic phenotyping provides reproducible and clinically meaningful information for risk stratification and treatment-response assessment. Clinical translation will also require transparent and externally validated analytical methods, including AI-based approaches, together with real-world evaluation of feasibility, accessibility, cost-effectiveness, and integration into existing clinical workflows.
