# Supplementary material for: Heart rate dynamics in polyendocrine metabolic ovarian syndrome: from heterogeneity to wearable digital phenotyping
Source: Front Endocrinol (Lausanne). 2026 Sep 22;17:1951746. doi: 10.3389/fendo.2026.1951746 (PMC13639433; doi:10.3389/fendo.2026.1951746)
Supplement: Supplementary file 1 [file DataSheet1.pdf]

Supplementary Material

Scope note. These tables are designed as a concise cross-disciplinary reference for the accompanying review. They summarize the terminology, computational definitions, and reporting considerations most relevant to the proposed framework. They do not establish PMOS/PCOS-specific clinical thresholds. Final analytic reports should also state software, version, preprocessing rules, recording duration, and signal-quality criteria.

Supplementary Table S1. Glossary of key terms and abbreviations

Terms are grouped by conceptual role. Definitions are intentionally concise and should be read together with the methodological cautions in Supplementary Table S2.

Category	Term / abbreviation	Definition	Interpretation or use in this review
**Disease context**	PMOS	Polyendocrine metabolic ovarian syndrome	New consensus name for PCOS; use PMOS/PCOS when discussing the updated disease concept.
**Disease context**	PCOS	Polycystic ovary syndrome	Retain when describing the established literature that used the historical name.
**Disease context**	ANS	Autonomic nervous system	Integrates neural regulation with cardiovascular, metabolic, and reproductive physiology.
**Assessment**	HRV	Heart rate variability	Variation in consecutive cardiac intervals; an indirect marker of integrated cardiovascular autonomic modulation.
**Assessment**	MSNA	Muscle sympathetic nerve activity	Direct regional measure of sympathetic vasomotor outflow; informative but invasive and difficult to scale.
**Signal acquisition**	ECG	Electrocardiography	Reference signal source for precise R-peak detection and NN-interval extraction.
**Signal acquisition**	PPG	Photoplethysmography	Peripheral pulse-wave sensing used by many consumer wearables; useful for long-term trends but sensitive to motion and signal-quality variation.
**Signal acquisition**	RR interval	Time interval between consecutive ECG R peaks	Includes all detected beats unless further filtered.
**Signal acquisition**	NN interval	Interval between consecutive normal sinus beats	Core input for HRV and higher-order heart rate dynamics analysis after artifact and ectopic-beat handling.

Category	Term / abbreviation	Definition	Interpretation or use in this review
**Signal acquisition**	SQI	Signal-quality index	Metric or rule set used to identify usable signal segments before feature extraction.
**Conventional HRV**	SDNN	Standard deviation of NN intervals	Global variability-amplitude measure; strongly dependent on recording duration.
**Conventional HRV**	RMSSD	Root mean square of successive NN-interval differences	Short-term variability measure commonly interpreted as vagal-related when recording conditions are controlled.
**Conventional HRV**	NN50 / pNN50	Count / percentage of successive NN-interval differences greater than 50 ms	Threshold-based time-domain measures commonly interpreted as vagal-related.
**Conventional HRV**	VLF	Very-low-frequency power	Spectral component below 0.04 Hz; interpretation is limited in short recordings.
**Conventional HRV**	LF	Low-frequency power	Spectral power from 0.04 to 0.15 Hz; influenced by multiple physiological processes and not a direct sympathetic measure.
**Conventional HRV**	HF	High-frequency power	Spectral power from 0.15 to 0.40 Hz; strongly influenced by respiration and commonly interpreted as vagal-related.
**Conventional HRV**	LFnu / HFnu	Normalized low- / high-frequency power	Relative spectral measures; report the normalization convention and avoid one-to-one autonomic interpretation.
**Conventional HRV**	LF/HF ratio	Ratio of low- to high-frequency power	Context-dependent descriptor; should not be treated as a direct measure of sympathovagal balance.
**Rhythm structure**	HRF	Heart rate fragmentation	Family of metrics describing beat-to-beat microstructural discontinuity in NN-interval dynamics.
**Rhythm structure**	Inflection point	Transition point at which NN-interval change reverses direction or changes to or from zero	Building block for HRF metrics.

Category	Term / abbreviation	Definition	Interpretation or use in this review
**Rhythm structure**	Acceleration / deceleration segment	Continuous run of NN-interval changes in one direction	Shorter segments indicate more fragmented directional organization.
**Rhythm structure**	PIP	Percentage of inflection points	Higher values indicate more frequent directional changes.
**Rhythm structure**	IALS	Inverse of the average length of acceleration / deceleration segments	Higher values indicate shorter average segment length and greater fragmentation.
**Rhythm structure**	PSS	Percentage of NN intervals in short segments	Higher values indicate a greater contribution of short segments.
**Nonlinear dynamics**	Poincare plot	Scatter plot of NN_i versus NN_(i+1)	Visualizes successive-interval geometry and local correlation structure.
**Nonlinear dynamics**	SD1 / SD2	Short- / long-axis descriptors of Poincare-plot dispersion	Geometric descriptors of short- and longer-term variability components.
**Nonlinear dynamics**	ApEn	Approximate entropy	Quantifies pattern irregularity; includes self-matches and depends on data length and parameter settings.
**Nonlinear dynamics**	SampEn	Sample entropy	Quantifies pattern irregularity without counting self-matches; depends on data length and parameter settings.
**Nonlinear dynamics**	MSE	Multiscale entropy	Evaluates entropy across coarse-grained temporal scales.
**Nonlinear dynamics**	DFA	Detrended fluctuation analysis	Characterizes scale-related correlation structure using a scaling exponent.
**Regulatory dynamics**	PRSA	Phase-rectified signal averaging	Aligns anchor-centered NN segments and averages them to reveal quasi-periodic acceleration- or deceleration-related patterns.
**Regulatory dynamics**	DC	Deceleration capacity	PRSA-derived descriptor of deceleration-related regulatory dynamics; more closely associated with vagally mediated deceleration.

Category	Term / abbreviation	Definition	Interpretation or use in this review
**Regulatory dynamics**	AC	Acceleration capacity	PRSA-derived acceleration-related regulatory descriptor; not a direct measure of isolated sympathetic activity.
**Monitoring**	Digital phenotyping	Longitudinal characterization of physiological patterns using digital measurements	Supports context-aware tracking across sleep, activity, diurnal timing, and reproductive context.

Supplementary Table S2. Computational definitions and reporting considerations

Notation. NN_i denotes the i-th normal-to-normal interval; N denotes the number of NN intervals analyzed. Operational definitions should be implemented consistently across participants. Where software packages differ, report the package, version, and parameter settings.

S2A. Conventional time- and frequency-domain HRV

Metric	Definition or formula	Report these inputs	Interpretation and caveats	Source
DeltaNN_i	$\Delta \mathrm{NN}_i = \mathrm{NN}_i - \mathrm{NN}_{i-1}$	NN filtering rules; units (ms); recording duration	Basic successive-difference sequence used by RMSSD, pNN50, and HRF calculations.	[R1]
SDNN	$\mathrm{SDNN} = \sqrt{\frac{\sum_{i=1}^{N} (\mathrm{NN}_i - \overline{\mathrm{NN}})^2}{N-1}}$	Recording duration; segment selection; artifact handling	Global variability-amplitude measure. Do not compare values from substantially different recording durations without qualification.	[R1]
RMSSD	$\mathrm{RMSSD} = \sqrt{\frac{\sum_{i=2}^{N} (\Delta \mathrm{NN}_i)^2}{N-1}}$	Recording context; respiration if available; artifact handling	Short-term variability measure commonly interpreted as vagal-related under standardized conditions.	[R1]
NN50 / pNN50	$\mathrm{NN50} = \sum_{i=2}^{N} I(\lvert \Delta \mathrm{NN}_i \rvert > 50\ \mathrm{ms})$ $\mathrm{pNN50} = 100 \times \frac{\mathrm{NN50}}{N-1}$	Threshold convention; segment duration; artifact handling	Threshold-based short-term measures commonly interpreted as vagal-related.	[R1]

Metric	Definition or formula	Report these inputs	Interpretation and caveats	Source
VLF, LF, HF power	$$P_B = \int_{f_{\text{low}}}^{f_{\text{high}}} S_{\text{NN}}(f)df$$ *Common adult bands: LF 0.04-0.15 Hz; HF 0.15-0.40 Hz. For long recordings, VLF is commonly defined as 0.0033-0.04 Hz; state the convention used.*	Spectral method; sampling; segment duration; detrending; respiration; band convention	HF is respiration-sensitive. LF has mixed physiological determinants. VLF interpretation is limited in short recordings.	[R1]
LFnu / HFnu	$$\text{LFnu} = 100 \times \frac{\text{LF}}{\text{LF} + \text{HF}}$$ $$\text{HFnu} = 100 \times \frac{\text{HF}}{\text{LF} + \text{HF}}$$ *This is a common convention; state the denominator explicitly.*	Normalization convention; respiration; spectral method	Relative measures; avoid direct attribution to isolated sympathetic or parasympathetic activity.	[R1, R10]
LF/HF ratio	$$\frac{\text{LF}}{\text{HF}}$$	Respiration; posture; recording context; spectral method	Should not be treated as a direct or isolated measure of sympathovagal balance.	[R10]

S2B. Heart rate fragmentation (HRF)

HRF is calculated from directional changes in the NN-interval series (see DeltaNN_i in S2A). An inflection point occurs when the direction of change reverses or changes to or from zero. An acceleration or deceleration segment is a continuous run of intervals changing in one direction.

Metric	Definition or formula	Report these inputs	Interpretation and caveats	Source
PIP	$$\text{PIP} = 100 \times \frac{N_{\text{IP}}}{N_{\text{NN}}}$$ *N_IP: number of inflection points; N_NN: number of NN intervals assessed.*	NN filtering; treatment of ties; recording duration; exact implementation	Higher PIP indicates more frequent directional changes in NN-interval dynamics.	[R2]
IALS	$$\text{IALS} = \frac{1}{\bar{L}}$$ *L-bar: mean length of acceleration / deceleration segments.*	Segment-definition implementation; NN filtering	Higher IALS indicates shorter average segments and greater fragmentation.	[R2]

Metric	Definition or formula	Report these inputs	Interpretation and caveats	Source
PSS	$$\mathrm{PSS} = 100 \times \frac{N_{\mathrm{short}}}{N_{\mathrm{NN}}}$$ *In the original method, short segments are defined as the complement of segments containing three or more NN intervals.*	Short-segment rule; NN filtering; recording duration	Higher PSS indicates a larger contribution of short directional segments.	[R2]

S2C. Nonlinear and geometric heart rate dynamics

Metric	Definition or formula	Report these inputs	Interpretation and caveats	Source
Poincare plot	$$(\mathrm{NN}_i, \mathrm{NN}_{i+1})$$ *Plot NN_i on the x-axis against NN_(i+1) on the y-axis.*	Segment duration; artifact handling; visualization settings	Visual geometric representation of successive-interval organization. Do not interpret it as HRF.	[R3]
SD1	$$\mathrm{SD1} = \sqrt{\frac{1}{2}\,\mathrm{Var}(\Delta \mathrm{NN}_i)}$$	Units; segment duration; artifact handling	Short-axis Poincare dispersion; closely related to successive-difference variability.	[R3]
SD2	$$\mathrm{SD2} = \sqrt{2\,\mathrm{Var}(\mathrm{NN}_i) - \frac{1}{2}\,\mathrm{Var}(\Delta \mathrm{NN}_i)}$$	Units; segment duration; artifact handling	Long-axis Poincare dispersion; reflects longer-term scatter within the analyzed segment.	[R3]
ApEn	$$\mathrm{ApEn}(m, r, N) = \Phi^m(r) - \Phi^{m+1}(r)$$	Embedding dimension m; tolerance r; N; normalization of r	Pattern-irregularity estimate that includes self-matches. Values depend on data length and parameter selection.	[R4]
SampEn	$$\mathrm{SampEn}(m, r, N) = -\ln\left(\frac{A}{B}\right)$$ *B counts matching template pairs of length m; A counts matches retained after extension to m + 1. Self-matches are excluded.*	Embedding dimension m; tolerance r; N; normalization of r	Pattern-irregularity estimate. Cross-study comparisons require harmonized parameters and preprocessing.	[R5]
MSE	$$y_j^{(\tau)} = \frac{1}{\tau} \sum_{i=(j-1)\tau+1}^{j\tau} x_i$$	Entropy estimator; tau range; m; r; data length; coarse-graining rule	Characterizes scale-dependent complexity. Reliable interpretation	[R6]

Metric	Definition or formula	Report these inputs	Interpretation and caveats	Source
	$$\mathrm{MSE}(\tau) = \mathrm{SampEn}(y^{(\tau)})$$		requires adequate data length at each scale.	
DFA alpha	$$F(n) \propto n^{\alpha}$$ *Estimate alpha from the slope of log F(n) versus log n after integration and local detrending.*	Scale range; alpha_1 / alpha_2 convention; detrending order; data length	Scaling descriptor of correlation structure. Avoid universal thresholds without matched analytic settings.	[R7]

S2D. PRSA-derived regulatory dynamics

Metric	Definition or formula	Report these inputs	Interpretation and caveats	Source
PRSA curve X(k)	$$X(k) = \frac{1}{M}\sum_{m=1}^{M} \mathrm{NN}_{a_m+k}$$ *a_m denotes the m-th acceleration or deceleration anchor.*	Anchor rule; window length L; averaging scale s; NN filtering	Reveals acceleration- or deceleration-related quasi-periodic structure despite noise and nonstationarity.	[R8, R9]
DC	$$\mathrm{DC} = \frac{X(0) + X(1) - X(-1) - X(-2)}{4}$$ *Shown for averaging scale s = 2 and calculated from deceleration-anchor PRSA.*	Anchor rule; L; s; recording duration; NN filtering; units (ms)	Descriptor of deceleration-related regulatory dynamics. More closely associated with vagally mediated deceleration, but not a direct neural recording.	[R8, R9]
AC	$$\mathrm{AC} = \frac{X(0) + X(1) - X(-1) - X(-2)}{4}$$ *Calculated from acceleration-anchor PRSA. Under common conventions, AC is negative.*	Anchor rule; L; s; sign convention; recording duration; NN filtering	Acceleration-related regulatory descriptor; not a direct measure of isolated sympathetic activity.	[R9]

Method sources

[R1] Task Force of the European Society of Cardiology and the North American Society of Pacing and Electrophysiology. Heart rate variability: standards of measurement, physiological interpretation, and clinical use. European Heart Journal. 1996;17:354-381.

[R2] Costa MD, Davis RB, Goldberger AL. Heart rate fragmentation: a new approach to the analysis of cardiac interbeat interval dynamics. Frontiers in Physiology. 2017;8:255.

[R3] Brennan M, Palaniswami M, Kamen P. Do existing measures of Poincare plot geometry reflect nonlinear features of heart rate variability? IEEE Transactions on Biomedical Engineering. 2001;48:1342-1347.

[R4] Pincus SM. Approximate entropy as a measure of system complexity. Proceedings of the National Academy of Sciences. 1991;88:2297-2301.

[R5] Richman JS, Moorman JR. Physiological time-series analysis using approximate entropy and sample entropy. American Journal of Physiology-Heart and Circulatory Physiology. 2000;278:H2039-H2049.

[R6] Costa M, Goldberger AL, Peng CK. Multiscale entropy analysis of complex physiologic time series. Physical Review Letters. 2002;89:068102.

[R7] Peng CK, Havlin S, Stanley HE, Goldberger AL. Quantification of scaling exponents and crossover phenomena in nonstationary heartbeat time series. Chaos. 1995;5:82-87.

[R8] Bauer A, Kantelhardt JW, Barthel P, et al. Deceleration capacity of heart rate as a predictor of mortality after myocardial infarction: cohort study. The Lancet. 2006;367:1674-1681.

[R9] Schneider R, Bauer A, Kantelhardt JW, Barthel P, Schmidt G. Phase-rectified signal averaging for the detection of quasi-periodicities in electrocardiogram. IFMBE Proceedings. 2007:38-41.

[R10] Billman GE. The LF/HF ratio does not accurately measure cardiac sympatho-vagal balance. Frontiers in Physiology. 2013;4:26.
